# Transverse Right Ventricle Strain and Strain Rate Assessed by 2-Dimensional Speckle Tracking Echocardiography in Dogs with Pulmonary Hypertension

**DOI:** 10.3390/vetsci7010019

**Published:** 2020-02-07

**Authors:** Domenico Caivano, Mark Rishniw, Francesco Birettoni, Vasilica-Flory Petrescu, Francesco Porciello

**Affiliations:** 1 Department of Veterinary Medicine, University of Perugia, Via San Costanzo 4, 06126 Perugia, Italy; florypetrescu@hotmail.com (V.-F.P.); francesco.porciello@unipg.it (F.P.); 2 Department of Clinical Sciences, College of Veterinary Medicine, Cornell University, Ithaca, NY 14853, USA; mr89@cornell.edu

**Keywords:** echocardiography, right ventricular function, cardiac, canine

## Abstract

Right ventricular (RV) strain analysis using 2-dimensional speckle tracking echocardiography (2D STE) permits practitioners to assess regional and global deformation of the myocardium. Recently, assessment of the longitudinal right ventricle (RV) strain and strain rate using 2D STE has been reported in dogs. Although longitudinal deformation is the dominant component of the RV systole, RV myocardial fibers of the superficial layer are oriented circumferentially and these contribute to the RV pump function (radial deformation). Because this strain component has not been investigated in dogs, we have assessed radial RV strain and strain rate using 2D STE in healthy dogs and dogs with pulmonary hypertension (PH). We have recruited 74 dogs (40 healthy dogs and 34 dogs with PH) in which we have analyzed radial RV free wall strain and strain rate using Xstrain^TM^ software. We have used the left apical 4-chamber view optimized for the RV for analysis of the radial strain and strain rate variables (Xstrain^TM^ software denoted radial strain as “transverse”). Seven dogs were excluded during the analysis for low quality images. Transverse strain and strain rate obtained in healthy dogs showed no relationship with heart rate, body weight or age (r^2^ < 0.09 and *p* > 0.05 for all variables). Moreover, no relationship between transverse strain/strain rate variables and left atrial-to-aortic ratios was observed (r^2^ < 0.06 and *p* = 0.2, for both). Transverse strain and strain rate obtained in dogs with PH, showed weak negative relationships with tricuspid regurgitation velocity (r^2^ < 0.25 and *p* = 0.006, for both). Transverse RV strain and strain rate using 2D STE is feasible in most dogs and decrease with worsening of the PH, but these advanced echocardiographic indices do not help in identifying dogs with PH.

## 1. Introduction

Echocardiographic assessment of the right ventricle (RV) is the most practical method for evaluating the RV morphology and function. Although complex anatomy of the RV makes the evaluation of this cardiac chamber a challenge, several echocardiographic indices have been investigated in dogs [1,2,3,4,5,6,7,8,9,10,11]. Repeatability and reference intervals for these indices have been evaluated but most of these echocardiographic indices only provide an assessment of regional function of the RV (apex, inflow or outflow tract) [1,2,3,4,5,6,7,8,9,10,11]. In humans, it is recommended to evaluate right ventricular (RV) morphology and function using multiple echocardiographic indices, including tricuspid annulus plane systolic excursion, fractional area change, RV Tei index, tricuspid annular velocity by tissue Doppler imaging and RV longitudinal strain by speckle-tracking echocardiography [12,13].

An advanced imaging modality, such as 2-dimensional speckle tracking echocardiography (2D STE), was demonstrated able to assess regional and global myocardial deformation by providing strain (change in percentage from the original dimension) and strain rate (velocity of this change) values. This method allows investigators to evaluate RV function without being influenced by geometric assumptions or angle dependence [14]. Right ventricular deformation analysis using STE has focused on assessing longitudinal strain and strain rate variables in dogs [1,7,8]. However, RV contraction is also characterized by inward movement of the RV free wall; this component of the RV contraction pattern, called radial or transverse deformation, has been evaluated using 2D STE in few reports [15,16,17].

To the best of the authors’ knowledge, no studies have investigated the transverse deformation of the RV in dogs. Therefore, we assessed the ability of measuring transverse RV strain and strain rate via STE in dogs. We also assessed whether transverse RV strain and strain rate could identify dogs with PH. Finally, we sought to evaluate the intra- and interobserver variability of measuring transverse RV strain and strain rate via STE in dogs.

## 2. Materials and Methods

We prospectively included in the study healthy dogs and dogs with PH presented at the Cardiology Service of the Veterinary Teaching Hospital of Perugia University. Healthy dogs of various breeds and sizes, aged >1 year, were recruited from the students, staff and owners. Dogs were considered healthy based on an unremarkable history, complete physical evaluation, and conventional transthoracic echocardiography. Dogs with PH included in the study were defined by a measurement of a tricuspid regurgitation (TR) velocity jet >3 m/Section [18]. Dogs with persistent non-sinus arrhythmias, dogs with congenital cardiac disease or other acquired diseases except for myxomatous mitral valve disease (MMVD) or PH, were excluded. Dogs receiving cardiac medications were not excluded. Owners of healthy dogs provided informed consent for the echocardiographic imaging. No client consent was sought in dogs presenting for the echocardiographic evaluation, because no additional stress was imposed on dogs to obtain the images used in this study.

### 2.1. Standard Echocardiography

Conventional echocardiography was performed by the same echocardiographer (D.C.) using an ultrasound machine (MyLab Class C, Esaote, Genova, Italy) equipped with multifrequency 1–11 MHz phased-array transducers. All dogs underwent an echocardiographic examination without sedation and gently restrained in lateral recumbency with continuous electrocardiographic recording. Two-dimensional, M-mode and Doppler echocardiography was performed using recommended right and left parasternal imaging planes [19,20]. Conventional M-mode echocardiographic measurements were compared with the reported reference ranges [21]. Left atrial-to-aortic ratios (LA: Ao) were obtained from the right parasternal short axis view using 2-dimensional imaging and aortic measurements were timed at the onset of diastole, immediately after closure of the aortic valve [22,23].

### 2.2. Speckle Tracking Echocardiography

**Figure 1:**
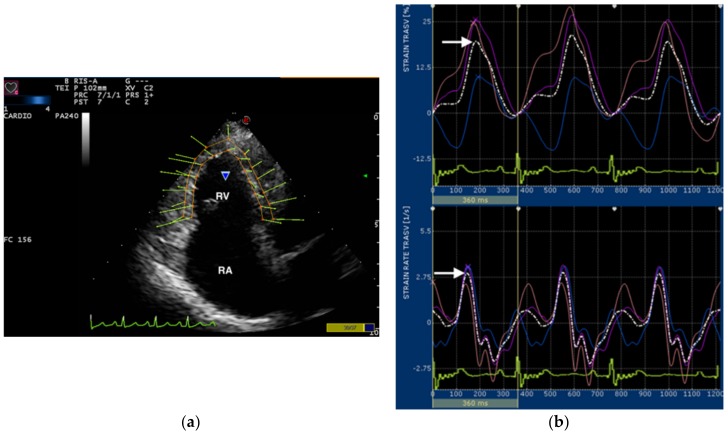
(**a**) Snapshot of 2-dimensional speckle tracking analysis from left apical 4-chamber view optimized for visualization of the right ventricle (RV). Thirteen points delimit and follow the endocardial and epicardial border of the RV frame by frame during entire cardiac cycle. The green arrows represent the vectors of each point and their direction of displacement. RV, right ventricle; RA, right atrium; (**b**) Snapshot of transverse strain (upper) and strain rate (lower) curves of the RV free wall. The software divided the entire RV into 6 segments, each containing 3 points (1 or 2 points were shared between adjacent segments). Tree segments corresponding to the interventricular septum were excluded and only three segments of RV free wall (colored line) were used for measurements. The software also generates an average value (dotted line) of the transverse RV strain and strain rate. The snapshot shows strain and strain rate curves over time in relation to the electrocardiogram (bottom). Only the systolic peak (arrows) of the transverse strain and strain rate of the RV free wall were used in this study.

Images of the RV for 2D STE were obtained from the apical 4-chamber view optimized for the RV visualization by multifrequency 1–11 MHz phased-array probes and three cineloops (at least three beats for each) were acquired for off-line analysis. All cineloops were analyzed by one experienced examiner (D.C.) using the Xstrain^TM^ software (Esaote, Genova, Italy), which allows semiautomated analysis of speckle-based strain. A frame with an optimally visualized endocardial and epicardial borders were selected. The software automatically traced 10 equidistant lines among 3 starting points fixed by the operator (2 at the tricuspid valve annulus and 1 at the RV apex). The operator fixed 10 points along the endocardial border and 13 equidistant points delimited RV endocardial border. Similarly, 13 equidistant points delimiting epicardial border of the RV free wall and interventricular septum were fixed (Figure 1). If automated tracking was suboptimal, the points were manually adjusted. Based on these points, the software generated 13 curves of transverse strain and strain rate. After this, the software divided the entire RV into 6 segments, each containing 3 points (1 or 2 points were shared between adjacent segments), and generated an average value of the radial strain and strain rate (Figure 1). The radial RV deformation analyzed from the apical view was denoted “transverse” by the system. Therefore, we refer to the radial strain and strain rate as the transverse strain and strain rate throughout the remainder of this manuscript. Only average value of the maximal systolic peak of the RV free wall transverse strain and strain rate were analyzed (Figure 1). The mean value of three consecutive cardiac cycles in sinus rhythm for each variable was obtained and used for statistical analysis.

To assess the intra- and inter-observer measurement variability, six echocardiograms of six different dogs were randomly selected for repeated measurements by the same observer (D.C.) and by a second observer (V.P.). Intraobserver measurement variability was evaluated by having each observer trace and analyzes the same cardiac cycles on two different days. Interobserver measurement variability was evaluated by having the two observers independently trace and analyze the same cardiac cycles.

### 2.3. Statistical Analysis

We first examined whether transverse RV strain and strain rate were different between healthy dogs and dogs with PH. We divided dogs with PH into three levels of severity (mild, moderate, severe), based on the following criteria (mild = tricuspid regurgitation (TR) velocity >3.1 m/s; moderate = TR velocity between 3.5 and 4.5 m/s; severe = TR velocity >4.5 m/s). We compared all groups with Kruskal–Wallis tests, with pairwise comparisons using the Conover method to identify differences between groups. To assess the diagnostic utility of transverse RV strain and strain rate in identifying dogs with PH, we performed several receiver operating characteristic curve analyses.

We next examined relationships of transverse strain and strain rate values with heart rate (average of three cycles recorded during the STE acquisition), age and bodyweight in healthy dogs, and with TR velocity and LA:Ao in dogs with PH by univariable linear regression after confirming that major assumptions of linear regression were not violated.

Finally, we examined inter- and intraobserver variability by calculating the percent difference between measurements by the same observer and between observers, as previously reported [24,25].

All analyses were performed using commercial statistical software (MedCalc Statistical Software version 19.1.5 (MedCalc Software bv, Ostend, Belgium; https://www.medcalc.org; 2020).

## 3. Results

A total of 74 dogs were initially evaluated, but seven dogs (three healthy and four dogs with PH) were excluded because of poor speckle tracking for low image quality, resulting in 67 dogs (37 healthy and 30 dogs with PH) that were finally available for analysis. Clinical characteristics for dogs included in the study are presented in Table 1.

We identified the cause of the PH as either post-capillary PH (secondary to moderate to severe MMVD) in 16/30 dogs or pre-capillary PH (presence of heartworm disease or identifiable primary pulmonary disease with unrelated to left-sided heart disease or with mild MMVD) in 14/30 dogs.

In all dogs, transverse RV strain curves were characterized by an increase in strain percentage during ventricular systole and a rapid decrease approaching zero during ventricular diastole (Figure 1). All average curves of transverse RV strain rate showed a positive wave during ventricular systole and 2 negative waves during ventricular diastole (Figure 1).

**Figure 2:**
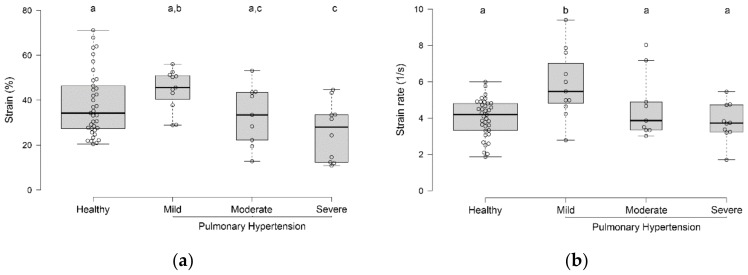
Box-and-whisker plots of transverse strain (**a**) and strain rate (**b**) values in healthy dogs and dogs with various severities of pulmonary hypertension. Groups with different superscripts (a, b, c) differed from each other.

Healthy dogs had higher strain values than dogs with severe PH (*p* = 0.02; Figure 2a). Dogs with mild PH had higher strain values than dogs with moderate or severe PH (*p* = 0.02; Figure 2a). However, only dogs with mild PH had strain rates higher than all other groups (*p* = 0.02; Figure 2b).

Based on the results obtained in the initial analyses, we further evaluated the diagnostic utility of strain (but not strain rate) in identifying dogs with PH. We performed 3 analyses: healthy vs. all dogs with PH, healthy dogs and dogs with mild PH vs. dogs with moderate and severe PH, and all dogs with less-than-severe PH vs. dogs with severe PH. Strain could differentiate dogs with at least moderate PH from healthy dogs or dogs with mild PH (*p* = 0.01), but displayed poor test performance (Area under the curve = 0.69). This did not improve substantially when limiting the analysis to dogs with severe PH (Area under the curve = 0.73).

**Figure 3:**
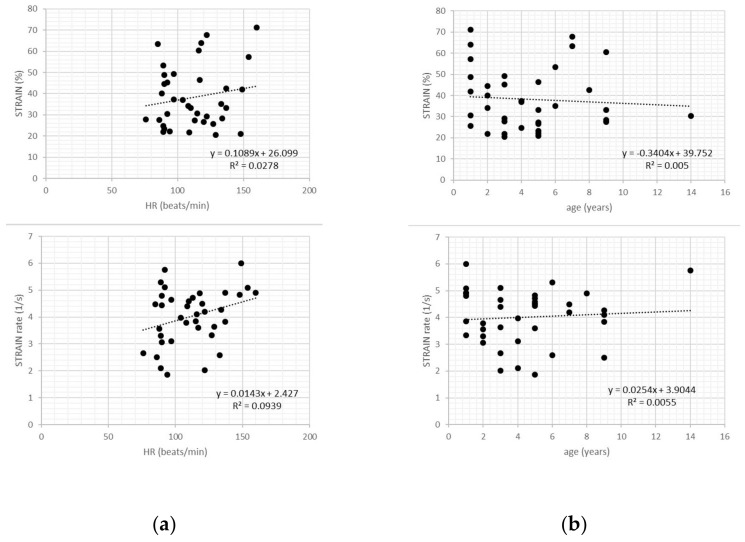

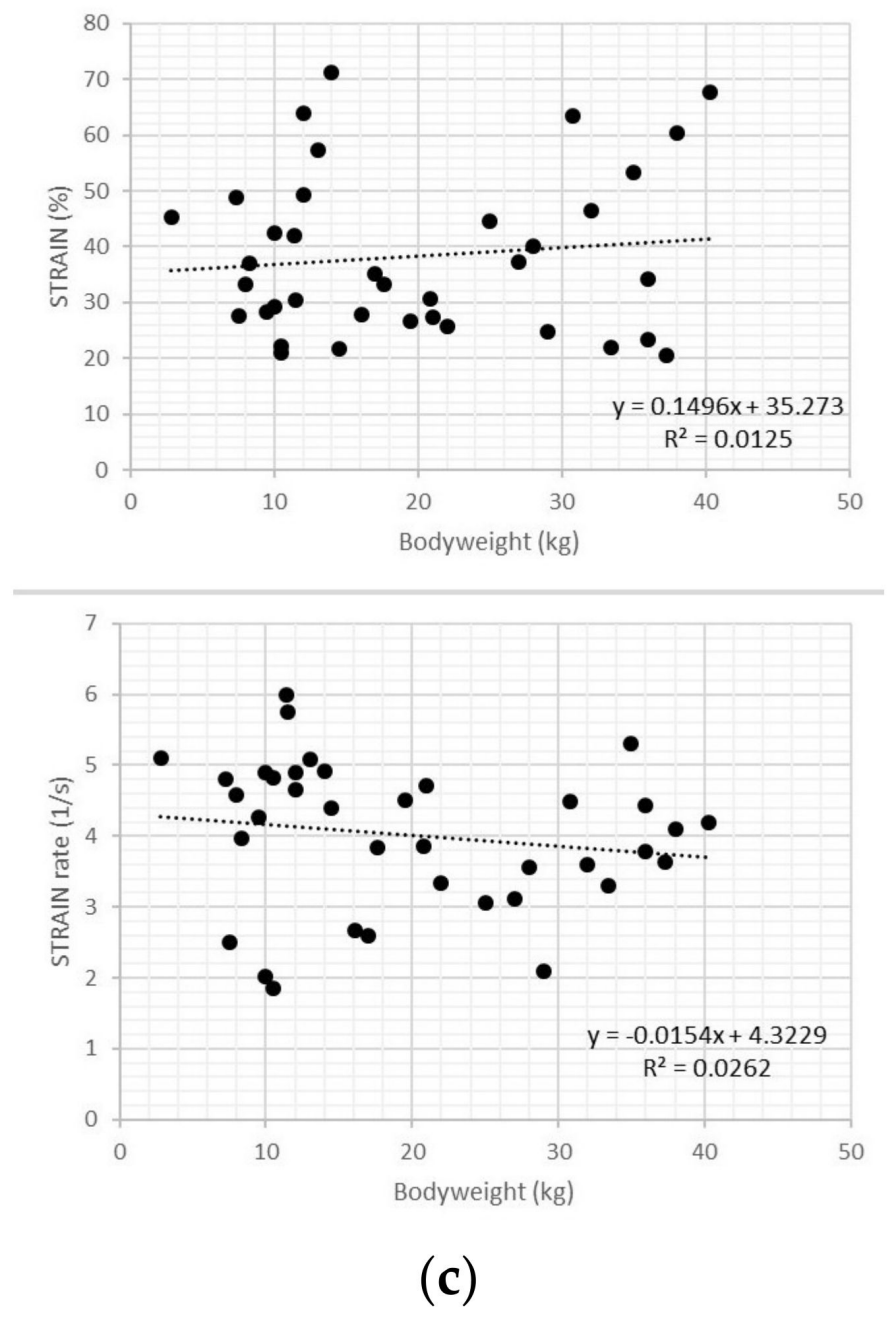
Scatter plots of transverse strain and strain rate values in healthy dogs showed no relationship with heart rate (**a**), age (**b**), and bodyweight (**c**).

In healthy dogs, transverse strain and strain rate values showed no relationship with heart rate, body weight or age (all *p* > 0.05) (Figure 3).

**Figure 4:**
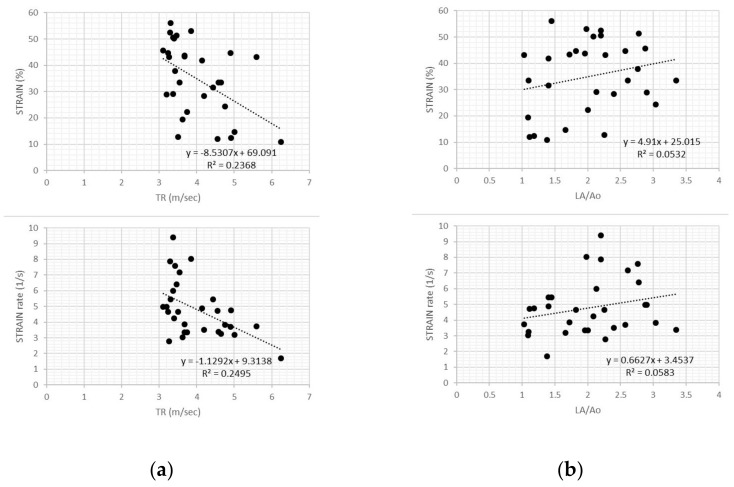
(**a**) Scatter plot of transverse strain and strain rate values in dogs with pulmonary hypertension showed weak negative relationships with tricuspid regurgitation velocity for both variables. TR, tricuspid regurgitation; (**b**) Scatter plot of transverse strain and strain rate values in dogs with pulmonary hypertension showed no relationship with left atrial size (*LA/Ao*) for both variables.

In dogs with PH, transverse strain and strain rate values showed weak negative relationships with TR velocity (r^2^ = 0.24 and r^2^ = 0.25, respectively; *p* = 0.006 for both variables), but no relationship with LA:Ao (r^2^ = 0.05 and r^2^ = 0.06, respectively; *p* = 0.2 for both variables) (Figure 4).

Intra- and inter-observer measurement variability for transverse strain values was 9.69% and 10.98%, respectively. For transverse strain rate values, intra- and inter-observer measurement variability was 20.65% and 22.78%, respectively.

## 4. Discussion

Our study shows that transverse RV strain and strain rate assessed by 2D STE can be obtained in most dogs, but occasionally, low quality image impedes acquisition. When applied to dogs with PH, transverse RV strain and strain rate values decrease with worsening of the PH, as estimated by TR velocity. However, these two echocardiographic indices do not help in identifying dogs with PH. Transverse strain showed low intra- and inter-observer measurement variability, while transverse strain rate showed high measurement variability both within and between observers.

We have assessed the RV function by estimating of the transverse RV strain and strain rate using STE in dogs with PH. Unlike the left ventricular free wall, the RV free wall shows a layer oriented longitudinally in the subendocardium and a second layer directed circumferentially in the subepicardium [26]. Most of echocardiographic indices reported in dogs have focused on the contraction of the longitudinal myocardial fibers, characterized by baso-apical shortening of the RV [1,2,4,6,7,8,9,10]. However, the circumferentially oriented superficial layer of myocardial fibers contributes to RV pump function. In humans, some investigators have reported that the multiple components involved in RV function pump varied under different conditions: RV myocardial fibers changed their spatial direction and were mostly circumferentially oriented in patients with RV pressure overload [27,28]. Moreover, the ratio of RV circumferential shortening to longitudinal RV shortening increased in patients with PH compared with normal subjects [29,30]. To our knowledge, no studies have investigated the radial deformation of the RV using conventional or advanced echocardiographic techniques in dogs. Our study demonstrated a negative relationship with the severity of PH, although the transverse strain and strain rate failed to accurately identify dogs with PH. Specifically, we found differences between healthy dogs and dogs with varying degrees of PH, but these groups overlapped sufficiently to render the measurements incapable of reasonably discriminating even dogs with severe PH from dogs with less-than-severe PH. Considering previous studies in humans [29,30], we speculate that further studies comparing the contraction of the RV longitudinal and circumferential fibers using STE, could clarify the change in the RV systolic function in dogs with PH.

We found no association between transverse RV strain and strain rate values and age, body weight or heart rate in healthy dogs. Our findings are somewhat consistent with previous studies assessed radial left ventricular strain and strain rate in dogs [31]. For age and body weight, our results are similar to previous study [31]. However, our healthy dogs did not show a normal (or even large) age distribution (only one dog was more than nine years old); therefore, we cannot exclude age-associated cardiac changes in healthy geriatric dogs. However, our results differ from those of a previous study [31] with regards to associations with heart rate. This could be due to different study populations, or software; however, we cannot exclude a different influence of heart rate on the radial contraction between the two ventricles.

Similarly, we found no association between transverse RV strain and strain rate values and left atrial size in dogs with PH. Therefore, post-capillary hypertension, most commonly associated with large left atria and left-sided congestive heart failure, does not appear to affect transverse RV strain and strain rate. Previous studies have reported that RV function can be influenced by changes in loading conditions of the left ventricle in dogs with PH secondary to MMVD [6,8,9]. In these studies, the authors assessed the longitudinal RV function by echocardiographic indices (TAPSE and STE analysis), which could be influenced by increased translational movements of the mitral valve annulus in dogs with MMVD. This would not be expected to affect the transverse RV strain and strain rate. Moreover, in our study, post-capillary PH was diagnosed by echocardiography without pulmonary artery pressure measurement by cardiac catheterization. Thus, the possibility of under- or overestimation of the PH and coexistent pre-capillary PH cannot be completely excluded. Finally, dogs with PH received multiple medications, which could have affected the STE parameters. Further studies are needed to assess the influence that the pre-capillary or post-capillary PH could have on transverse RV strain and strain rate values.

Transverse strain measurements showed low intra- and interobserver measurement variability (approximately 10%, consistent with acceptable variability for many biological variables). However, transverse strain rate measurement showed high intra- and interobserver measurement variability (upward of 20%). As we have previously reported in our study of left atrial strain [24,25], the software automatically generates the curves from the traced points during cardiac cycle, so this high variability for some variables might be software dependent (no dedicated software currently exists for either atrial or RV strain and strain rate estimates).

We failed to obtain STE measurements in 7/74 dogs because of poor speckle tracking due to low quality image. We emphasize that acquisition of STE data requires an optimal echocardiographic visualization of the RV. The STE software needs to clearly visualize the epicardial border of the RV free wall to follow it and assess the transverse strain and strain rate. RV free wall visualization by modified left apical 4-chamber view can be suboptimal because this portion of RV is located close to the lung, even with views optimized for visualizing the RV. Moreover, thoracic conformation of dogs or cardiac remodeling in dogs with PH can influence the image quality and the ability of the software to follow the speckles of the epicardial border of the free wall. Finally, sonographer’s experience can influence the quality of the echocardiographic images and an appropriate learning curve is necessary before performing speckle tracking analysis.

## 5. Conclusions

In conclusion, this study demonstrates for the first time that transverse RV strain and strain rate can be obtained from the apical 4-chamber view optimized for the RV visualization using the Xstrain^TM^ software. Based on our results, the clinical utility of radial deformation analysis evaluated by STE to assess RV myocardial function appears limited, but additional studies should be pursued to clarify the different involvement of longitudinal and radial myocardial fibers in the RV remodeling secondary to PH in dogs.

## Figures and Tables

**Table 1:** Clinical and echocardiographic data for 67 dogs included in the study.

	Healthy Dogs	Dogs with PH
Number of dogs (male)	37 (20)	30 (16)
Age (years)	4 (1–14)	12 (5–17)
Body weight (kg)	17 (2.8–40.3)	9.4 (2.2–31)
Heart rate (bpm) LA: Ao	110 (76–160) 1.44 (1.1–1.68)	137 (86–208) 2.0 (1.0–3.3)
TR velocity (m/Section) Transverse RV Strain (%) Transverse RV Strain rate (1/s)	– 34.2 (20.5–71.1) 4.2 (1.9–6.0)	3.7 (3.1–6.2) 35.7 (10.9–56.0) 4.6 (1.7–9.4)

Data are presented as median (range) for age, body weight, heart rate, LA:Ao, TR velocity and transverse RV strain and strain rate variables. PH, pulmonary hypertension; LA, left atrium; Ao, aorta; TR, tricuspid regurgitation; RV, right ventricle.
